# A Preliminary Study on the Validity and Stability of Projective Methods: An Application of the Structural Approach of Social Representations with Traditional Mexican Cheeses

**DOI:** 10.3390/foods11243959

**Published:** 2022-12-07

**Authors:** Edgar Rojas-Rivas, Humberto Thomé-Ortiz, Angélica Espinoza-Ortega

**Affiliations:** Instituto de Ciencias Agropecuarias y Rurales (ICAR), Universidad Autónoma del Estado de México, Toluca 50000, Mexico

**Keywords:** stability and validity, traditional foods, Mexican cheeses, projective methods, consumer behavior

## Abstract

Projective methods are qualitative tools used to study food consumer behavior. In recent years, there has been an increase in studies that use these tools to analyze consumer behavior, particularly with the word association (WA) technique. However, one of the challenges in using these methods is the stability and validity of the data. This research aimed to obtain preliminary information on the stability and validity of the associations generated by consumers with the WA technique, using the structural approach of social representations. For this, two studies were carried out; for the first study, a face-to-face survey was carried out in which 89 consumers participated, who wrote the first words that came to mind with the stimulus “Aculco” on a ballot paper. For the second study, 122 consumers completed the same task as in the first study; however, the participants were recruited from an online survey. A random sample (n = 50) of both studies was selected to explore the stability and validity of the results. In the three study samples, the words were grouped into categories and analyzed through the structural approach of social representations. The frequency of mention of the identified categories was compared with the chi-square test and the average position (AP) and the Cognitive Salience Index (CSI) were calculated. Prototype maps were built to study the structure of the categories according to the central core and peripheral areas. Cluster analysis was performed to corroborate the structure of the representations. Finally, multiple factor analysis (MFA) was performed to determine the similarity of the results obtained from the three samples using the RV coefficient. No statistical differences (*p* > 0.05) were identified in most of the representations (n = 11) generated from the WA task. Furthermore, the representation “Cheese and dairy products” was positioned in the central core of the three maps. The APs and the CSIs of each representation were similar in the three study samples. The RV coefficient (≥0.80) indicated similarity in the representations obtained. Results of this research can be useful for future studies that attempt to compare the stability and validity of the information based on qualitative and more flexible methodologies. Some methodological implications related to the validity and stability of projective methods are discussed.

## 1. Introduction

Traditional foods are an essential part of any culture, since they reflect the way of eating in each society. In the case of Mexico, there is a vast amount of traditional foods that consumers recognize as part of the gastronomic heritage of each region in the country [1,2]. Furthermore, several studies on the perception, preferences, habits, and factors associated with the consumption of traditional foods have been carried out in Mexico with food products such as cheeses [3], traditional foods in general [1], products with a traditional/functional duality [4], traditional beverages [5], maize tortillas [6], among others. These products are associated with the know-how involved in their preparation or production, their origin, sensory characteristics, the occasions and festivities related to their consumption, and the gastronomic heritage they represent [1,7,8,9].

At the methodological level, traditional foods have been studied from different perspectives, from quantitative methods, such as hedonic tests, conjoint analysis, sorting tests, and surveys with attitudinal questionnaires, to more flexible qualitative methods, including focus groups and depth interviews, or projective methods, such as the WA technique [10]. In the case of the WA technique, it has been observed that “traditional foods” have been among the most studied food categories through this technique, allowing a rapid approach to the object of study, due to its ease of use and versatility [11]. 

The WA technique is part of a group of qualitative techniques known as projective methods [12]. These methods assume that by presenting an ambiguous and unstructured stimulus, such as a word, phrase or photo, the study subjects’ most personal beliefs or attitudes can be accessed, which could significantly influence their behaviors. Its origins date back to the beginning of the 20th century when different clinical tests were carried out based on this task [13]. Over time, its use has spread and become popular for studying consumer behavior and market research. However, one of the challenges in using this type of qualitative tool is the validity and stability of consumer responses [12,14,15]. In other words, the associations are stable and valid in repetitive studies or with different sample sizes and sociodemographic characteristics.

Another challenge involved with the stability and validity of associations is the process of grouping words into categories or dimensions of a higher hierarchy. Three experts generally carry out this process on the subject, who, through their interpretation, group the words into categories until reaching a consensus on the terms that conform to each category. Another aspect to highlight is the word grouping criterion, which, according to recent scientific evidence, suggests at least 5% in the frequency of the mentioning of words (although there are disparities in the word grouping processes in several studies). Finally, another of the most relevant challenges, which is of interest to this research, is the way of obtaining the responses (words, phrases, or comments), either from ballots used face-to-face or through online surveys [16]. When comparing the results of both methods of obtaining information, the stability and validity of the responses can be evidenced [11].

Most studies that have used the WA technique in consumer studies obtain information through ballots and online surveys; the latter method has been used in cross-cultural studies or in large geographic regions where it is desired to know the preferences or consumer perceptions quickly, without the need to invest time or considerable financial resources. In addition, other authors [12,16] suggest that online surveys provide reliable information from projective methods. Some other studies obtain information with the WA technique through questionnaires or face-to-face interviews, which implies using ballot papers and online surveys, that is, a combination of both types of sampling [17,18,19]. However, the objective of the studies mentioned above is not to study the stability and validity of the associations. To our knowledge, no research has been identified that analyzes the stability and validity of the information generated from the WA technique in a food context based on these two methods of obtaining information, ballot papers vs. online surveys.

On the other hand, the structural approach of the social representation theory can be instrumental in studying the stability and validity of consumer associations using the WA technique. This perspective is based on the fact that all the words, phrases, or ideas generated from an associative task can be structured in a prototype map that is differentiated according to their importance and cognitive hierarchy [20]. 

The structural approach of social representations is based on the central core theory [20], in which the knowledge or information that a population group possesses towards the object of representation is divided into the following two main areas: central core and peripheral areas. The central core represents the homogeneity of the object of representation, which is strongly marked by the historicity and identity of a social group, while the peripheral areas represent the heterogeneity towards the object of representation, with the following two main functions: to protect the core and to allow its operation over time. In addition, peripheral areas are more sensitive to incorporating new representations marked by trends and sociocultural changes [21].

One of the pioneering studies to use this approach in a food context was that of Rodrigues et al. [22]. They studied the concept of wine minerality among consumers and winemakers in France. According to these authors, for both groups, wine minerality is associated with “terroir.” Subsequent studies have used this approach to study the social representations of different food products or concepts, such as beer [23], foods with edible flowers [24], pulses [25], or concepts such as infant formula or breastfeeding [26]. However, this approach has not yet been used to study the stability and validity of associations generated by consumers towards different food products, such as traditional Mexican foods.

This research aimed to study the stability and validity of the information generated from the WA technique in two consumer studies. The data were obtained with paper ballots and an online survey using the structural approach of social representations. For this, a territory in Central Mexico was taken as a case study, characterized by the production of traditional cheeses (also known as “*Quesos de Aculco*”) that consumers highly recognized in the region. Furthermore, this territory belongs to a tourist program in Mexico called “*Pueblos mágicos*”, which began in 2001 and serves as a symbol of recognition of the territories that preserve the natural and material heritage in the country [27], since they are unique places in terms of historicity and authenticity, including the foods that are produced in these places.

### 1.1. Literature Review

#### 1.1.1. Factors That Influence Food Consumer Behavior

Food choice is a process in which consumers select, acquire, prepare, and consume food based on various intrinsic (sensory characteristics) or extrinsic aspects of food (external cues, such as labeling, packaging, and advertisements, among others) [28]. However, deciphering the food consumer behavior is a complex task, since the consumer not only bases his/her decisions on the intrinsic characteristics of the food, but other extrinsic aspects influence his/her consumption behavior, such as convenience, practicality, mood, perceived healthiness of food or labeling, among others [11,29].

In addition, food choice goes beyond fulfilling a physiological function in the human body; that is, food selection is made based on personal (age, gender, income and personality), biological (hunger and appetite), or environmental factors, such as culture, social, religious, and economic aspects [30]. Therefore, different models, including the Food Choice Questionnaire (FCQ), have captured the main factors that influence food consumption in different cultures [31,32,33], even with traditional foods [1,34]. Some authors have studied the personal traits of rejection of new foods or foods outside one’s diet (including traditional ethnic foods) [5,35,36,37] or those made with new food technologies [38]. Likewise, several studies have reported the changes in the consumption patterns of different populations in the world since the COVID-19 pandemic [39]. However, in recent years, the methodologies used to understand food consumer behavior have moved from quantitative instruments, such as questionnaires or validated scales, towards more flexible tools, such as open-ended questions, free comments, free listing or drawings, or projective methods [11]. This is because their application is more straightforward, requires less time, costs, human resources, and a more significant amount of information is obtained from these methods. 

#### 1.1.2. Projective Methods: The WA Technique

Projective methods show consumers’ personalities, beliefs, or attitudes toward an object of consumption (including food), which would be difficult to capture from questionnaires or scales. These methods allow more information on extrinsic and intrinsic factors that contribute to people’s consumption decisions to be obtained. Their use has increased markedly in recent years for studying food consumer behavior. One of the most remarkable projective methods is the free word association test (WA). This can be established from the number of publications that have used this projective technique in the last twenty years [11].

At a theoretical and methodological level, the WA technique is based on the expectancy-value theory, which establishes that the first ideas, associations or words that a consumer expresses towards a stimulus reflect their beliefs, which shape attitudes and behavior towards a consumer object [11,12,37]. In addition, other theoretical models have been used to explain and structure the associations that consumers generate from an exercise of this nature. In particular, the theory of social representations (TSR) has been used by numerous studies to structure a person’s words, including in the field of food consumer science. Social representations are based on the standard and non-institutionalized language that a person or social group use towards an object of representation. Therefore, it is possible to identify the social representations of an object of consumption with the WA technique [40].

The TSR establishes that all the information generated from an associative task is based on three dimensions. The first is “Information”, which refers to the sum of information or knowledge that a social group possesses toward an object of representation. The second is known as the “Field of Representation”. It allows the visualization of the hierarchy of representations, which is generally based on different indicators, such as the frequency of mentioning words and the AP of each representation. Finally, the third dimension is “Attitude”, which establishes the representations’ connotation and shapes consumers’ attitudes and behaviors. In other words, if the meaning of the representation is positive/negative, it may lead to acceptance/rejection behavior towards an object of consumption [2,11,21,22,23].

Furthermore, different approaches in conjunction with TSR have been used to understand food consumer behavior. One of them is the structural approach, in which a prototype map of four quadrants is generated to identify the representations’ central core and peripheral areas. Generally, the prototype map is designed considering each representation’s citation frequency (*Y*-axis) and AP (*X*-axis). Therefore, the representations in the central core can be identified and positioned in the map’s upper-left area, while the peripheral regions are found in the upper-right (first periphery) and lower quadrants of the map (left = contrasting elements zone; right = second periphery). As previously established, this approach has been used with different concepts or food products taken from the language of consumers, including cheeses [22,23,24,25,26,41]. 

#### 1.1.3. Cheese Consumer Behavior: Application of Projective Techniques and Other Sensory Tools

On the other hand, the application of the WA technique for the study of food consumer behavior has been carried out in various food categories [11,12], including traditional foods, alcoholic and non-alcoholic beverages, meat and seafood products, unfamiliar foods for consumers, and other food categories, including cheese and dairy products [42,43,44,45]. For example, Soares et al. [43] investigated the perception of consumers from the northeast and southeast regions of Brazil towards Coalho cheese, identifying 17 categories, which demonstrate that the form of preparation, its association with a cultural product from the northeast region, as an accompaniment of regional gastronomy products, as well as their sensory characteristics are among the most important factors in the perception of consumers. Judacewski et al. [44] identified the perceptions of consumers towards cheeses ripened with a white mold surface in Brazil, establishing that sensory characteristics are the terms that consumers mostly associate with these types of cheeses. Eldesouky et al. [45] studied the influence of packaging on consumer expectations towards cheese, establishing that the practicality of opening the product, as well as the size and the transparency of the package, are the characteristics that most influence consumer expectations. In addition, other methods such as diaries have been used to study sensory perception and consumer behavior toward Canastra cheese [46].

From the above, it can be established that various factors influence consumers’ perceptions, attitudes, and behavior toward cheeses. However, Katthab et al. [47] show that sensory aspects, such as color, flavor, texture, and appearance, are crucial in the consumer acceptability of cheeses that have undergone a maturation process. Likewise, the cited authors [47] review various sensory methodologies applied to consumers to understand the factors that influence the acceptability of cheeses, including hedonic tests, triangle tests, projective mapping, CATA questions and flash profiles. Bimbo et al. [48] reviewed different studies on the influence of personal characteristics, such as gender, age or psychological factors, on consumer attitudes toward dairy products with health benefits, including cheese. For example, these authors identified that female consumers, compared to males, presented more positive attitudes toward low-fat cheeses. Additionally, older consumers were more aware of the health-enhancing characteristics of some dairy products.

Regarding traditional cheeses in Mexico, information on consumer perception and behavior toward these products is still limited. Some works, such as that of Agudelo-López et al. [49], studied the sensory perception of consumers towards Ocosingo ball cheese, one of the 40 traditional Mexican cheeses that is still made using artisanal processes in the country. The authors evaluated this cheese (with different stages of maturation) in three gastronomic scenarios, establishing an association between the intrinsic aspects of the product (sensory characteristics) and consumption motivations. López-Díaz and Martínez-Ruíz [50] carried out a sensory profile of Chihuahua cheese (also known as Mennonite or Chester), relating the sensory characteristics evaluated by a panel of experts with consumer preferences. The authors established that high acidity, a salty and bitter taste, and high-fat content limit the selection of this type of cheese. 

In this brief review of the literature, some of the factors that influence food consumer behavior have been shown, as well as the use of projective methods for their study, addressing theoretical and methodological aspects of their application. Likewise, some applications of projective techniques have been identified to study cheese consumer behavior. Finally, the review has been contextualized with some examples that have tried to capture the perceptions or behaviors of consumers in Mexico towards traditional cheeses. However, the application of projective methods (particularly with the WA technique) has not been carried out to study the validity and stability of the associations using traditional cheeses made in a region in Central Mexico.

## 2. Materials and Methods

### 2.1. Participants

This research was carried out with two studies. For the first study, a face-to-face questionnaire with ballot papers was applied, with the participation of 89 consumers from different locations in the State of Mexico, the state with the largest population in Mexico. Regarding the sample composition, 58% were women, more than half of the sample were people between 26 and 45 years of age, and the majority (70.78%) were regular consumers of cheese (once or twice a week, they consume some cheese). The questionnaires were applied randomly from 1 to 30 October 2021. The only inclusion criterion is that the participants were available and interested in participating in the study.

The same questionnaire applied in the first study was designed on the Google Forms ® platform for the second study. For this task, 122 consumers participated; 59% were women and most were subjects between 17 and 35 years of age, and regular cheese consumers (60.65%). The survey was distributed on social networks such as Facebook and WhatsApp. Most of the participants came from different localities in the State of Mexico. However, 12 subjects were from other states, such as Jalisco, Guerrero, Puebla, Guanajuato, and Mexico City. Their responses were not omitted as they completed the survey.

To obtain insights into the stability and validity of the associations generated in the study area, regardless of the participants’ sociodemographic characteristics and frequency of consumption [11,12], only balanced samples were taken with the variable sex in the two studies (Table 1). Regarding age, educational level, and frequency of consumption, statistically significant differences were identified in both samples. There were more subjects who were 17 to 25 years old in the study with the online survey, while more people aged between 36 and 45 years were in the study with paper ballots. Regarding the educational level, there was a significantly higher number of participants with a low educational level in the survey using ballot papers. In contrast, more people with a high educational level were recruited in the online survey. Likewise, there was a significantly higher number of frequent consumers in the online survey than in the ballot paper survey. Table 1 details the sociodemographic characteristics and the statistical differences between the study samples.

### 2.2. Random Sample

To obtain more information about the stability and validity of the projective methods, it was decided to randomly select 25 participants from each study and identify if the representations (frequency of mentioning words, AP, and CSI) were stable and reliable in the three study samples. Participants were randomly selected based on their ID and each participant had the same probability of being selected. For this random sample, it was also important that the sample was different concerning some sociodemographic characteristics of the other two samples. This sample (n = 50) was made up of mostly women (68%), between 26 and 45 years of age (66%), with a high educational level (48%) and regular consumption of cheese (66%). Participants of this sample did not present statistical differences (*p* > 0.05) regarding gender and frequency of consumption compared to the other two samples, while regarding age and educational level, the sample was statistically different according to the chi-square test (*p* < 0.01).

This research was approved by the National Council of Science and Technology (Consejo Nacional de Ciencia y Tecnología, CONACYT) under the program “Estancias Posdoctorales Por México” and had the following registration number: BP-PA-20210513211515183-1115531. 

### 2.3. Survey Instrument 

The three authors of the study carried out the content validation of the instrument. They established by consensus the stimulus to be used to obtain the associations of consumers towards the object of study. It was decided to use the term “Aculco” to identify the influence of the territory represented by the traditional cheeses made in the region. A pilot test was applied with 15 subjects to identify the inconsistencies and interpretability of the instrument [51]. Likewise, four words were requested during the associative task as part of the structural approach of social representations [21,23,25].

All participants agreed to participate in the study and written consent was obtained. In addition, in both surveys, the subjects were informed that the data collected would be only used for academic purposes, following the protocols of confidentiality and protection of personal data. Then, for both studies, participants were asked to mention the first four words that came to mind with the stimulus “Aculco”.

For the first study, consumers were given a paper ballot with the following instructions: could you mention the first four words, phrases, or ideas that come to mind with the term “Aculco”? The task took between 5 and 10 min. Regarding the online survey, the stimulus used to obtain the participants’ associations was the following question: could you mention the first four words, ideas, or phrases that come to mind when you hear the word “Aculco”?

### 2.4. Data Analysis

#### 2.4.1. Word Grouping into Categories

All information from the three study samples was placed in an Excel database. The words obtained from the WA exercise were qualitatively analyzed for their subsequent grouping into categories, following the methodological processes established by other authors [52]. This process was carried out by three experts with at least five years of experience in exercises of this nature (WA tasks). Independently, each expert grouped the words into categories of higher hierarchy in both studies. The grouping of words was carried out carefully to avoid the loss of information and misinterpretation of the terms and to group them in the correct category. The experts used a Spanish dictionary during the word grouping [23]. This process was carried out with the random sample selected from both studies.

Once the categories/representations were formed, the three experts met to contrast their results and agreed on categories that emerged in each study. Categories/representations mentioned by 5% of the sample (at least in one study) were considered for further analysis. The chi-square test was used to determine if the frequency of mentioning specific words differed in the three samples for each of the representations selected to analyze the stability and validity. This analysis expects that no statistical differences will be observed in the frequency of mention in most categories/representations with the Chi-Square test (*p* > 0.05).

#### 2.4.2. Structural Approach of Social Representations

Subsequently, to contribute to the study of the stability and validity of the information obtained from projective methods, such as the WA technique, the structural approach of social representations was used [20], which allows for identifying the central core and peripheral areas with prototype maps of the following four zones: central core, first periphery, contrasting elements zone, and second periphery. Rodrigues et al. [22] introduced the structural approach of social representations to study representations of wine minerality among consumers and winemakers in France. Several studies have subsequently used it to analyze concepts or food products [23,24,25,26].

For this analysis, the position of each word within the categories/representations was respected, considering that the first word is the most important and the fourth the least, given that in this associative task, the participants were not asked to order the words according to their hierarchy or importance [53]. In addition, other authors [54] establish that the first word mentioned with the WA technique has the most special connection with the object of representation and influences attitudes and behaviors regarding food selection among consumers [11].

The frequency of mentioning specific words and the AP of each category were considered. Multiple responses regarding the frequency of the categories/representations by each participant were considered only once, taking into account their first mention and position [26]. In this sense, a prototype map was built for each study, including the survey with ballots (face-to-face), online survey, and the random sample. 

The prototype maps were constructed using a scatter plot, considering the importance of each category/representation on the *X*-axis and the frequency of mentioning specific words on the *Y*-axis (%). The cut-off points for each map axis were calculated following the methodological processes established by other authors [21,23,26]. All categories/representations were placed in descending order for the cut-off point on the *Y*-axis. The cut-off point was established when the difference between two consecutive categories was at its maximum value. For the *X*-axis, the importance of the words in the WA exercise was averaged, resulting in a theoretical cut-off point of 2.5 = (1 + 2 + 3 + 4)/4 [23]. From this analysis, it was expected that the representations would have a similar configuration in the prototype maps of social representations in the three study samples, which could contribute to the study of the stability and validity of the information obtained from the projective methods.

#### 2.4.3. Cognitive Salience Index (CSI)

To corroborate the results obtained from the analysis of social representations, the CSI was calculated following the methodological processes established by other authors [55,56]. The formula used to calculate the CSI is as follows:

CSI = F/N × AP

where F is the frequency of mention of each category, N is the total number of participants and AP is the average position of each category/representation. The CSI ranges between 0 and 1; if a representation obtains a value close to 1, it indicates cognitive importance towards the object of presentation, with a high frequency of mention and an AP close to the first word mentioned. For the representations of lower frequency, the AP is close to the last word mentioned and the CSI is close to 0. This index was calculated using the three databases, so the APs of the representations and the CSIs are also expected to be similar. In other words, the representations at a cognitive and attitudinal level between the territory and traditional foods are similar.

#### 2.4.4. Cluster Analysis

Cluster analysis was used to complement the previous analyses and study the configuration of the representations identified with the three samples. Therefore, three distance matrices were constructed, considering the positions of the categories identified (rows) by the study subjects (columns). For the first sample, a 12 × 89 matrix was constructed; for the second, a 12 × 122 matrix; and for the third, a 12 × 50 matrix. Subsequently, the distances were calculated using the Manhattan distances [57,58].

Cluster analysis was conducted to assess whether the social representations constructed from the WA task could be grouped into different or similar clusters. Hierarchical cluster analysis (HCA) was performed using Ward’s method as an agglomeration algorithm and a dendrogram established the resulting groups in the three study samples. From this analysis, it was expected that the representations would have a similar grouping in the clusters identified from the HCA. 

#### 2.4.5. Multiple Factor Analysis (MFA)

Given that in the three samples, the results for each of the representations were standardized concerning the frequency, the APs (1–4) and CSIs (0–1), MFA was used to identify if the results were similar in the study samples. MFA is a multivariate statistical technique that analyzes a series of observations from various blocks or sets of variables of a qualitative or quantitative nature. The main objective of the MFA is to combine different groups of variables that describe the same observations [59,60,61]. Therefore, MFA was used to identify if the configurations of the three blocks of variables obtained were similar concerning the representations analyzed with the WA technique. 

In this case, the observations introduced to the MFA were the social representations identified (n = 12) using the same block of variables, including relative frequency, AP and CSI. Therefore, the procedure used by other authors was followed [18,62], placing the social representations in the rows of the database, while the frequency, AP and CSI were in the columns, as reported in Table 2. Finally, the regression vector coefficient (RV) was used to measure the correlation of social representations from the three blocks or variables. The RV coefficient is a measure that ranges between 0 and 1. A value close to 1 of the RV coefficient indicates similarity in the configuration of the social representations [18]. All statistical analyses in this article were performed using the software XLSTAT 2014.5.03 (Addinsoft Corp., Paris, France).

## 3. Results

### 3.1. Influence of the Territory on the Social Representations of Traditional Mexican Cheeses

The first result of this study that must be highlighted is that the social representations identified (n = 12) in the three samples were the same, with “Cheese and dairy products”, “Pueblo mágico”, and “Territory” being the most frequently mentioned, which suggests that the representations obtained indicate stability and validity towards the object of representation (Table 2). It is also essential to highlight the contrasting of the results by the three experts who grouped the words into higher hierarchy categories/representations.

Appendix A describes the most relevant words that formed each representation of the online and paper ballot surveys. It should be noted that in most of the representations (n = 11), the frequencies were similar, since no statistical differences were observed with the chi-square test (*p* > 0.05). This result reinforces the stability and validity of the information obtained from the WA technique described above. In other words, similar results were obtained from repetitive studies with projective methods (Table 2).

From the qualitative descriptive analysis, it was observed that the category “Cheese and dairy products” refers to the fact that consumers recognize the territory from the production of these products; some of the words included in this representation were as follows: cheese, cheeses, dairy products, milk, cheese production, among others. Furthermore, the AP of this representation in the three samples suggests that it has the most remarkable connection with the object of representation, since it was the closest to the first word mentioned. Even the CSIs values were the highest in the three study samples (paper ballots = 0.425, online survey = 0.550, and random sample = 0.678 (Table 2)).

The second representation (Pueblo mágico) should be highlighted, which refers to the fact that the territory studied is part of a Mexican tourism program with the same name and includes localities or municipalities of historical importance in the country with attractions that show their national identity. In this representation, the APs were similar, highlighting the CSIs, which were almost identical in the three study samples. In the third representation (Territory), the APs were also similar, but not with the CSI, given that in the random sample and with the paper ballots, these were slightly higher (CSI = 0.123; 0.128, respectively), unlike in the online study (CSI = 0.099).

In the representation “Nature and landscape”, statistically significant differences were observed in the frequency of mention (*p* < 0.05), since it was a representation mentioned more in the sample with ballot papers than in the other two samples. In this representation, the words mentioned indicate that the territory has an exceptional landscape and nature. Concerning the representation “Tourist resource (waterfall)”, it can be found that it is a resource that the territory possesses and that it attracts people to visit the region. No statistical differences were observed in the frequency of mention. In addition, the APs were similar between all three samples; however, the CSI was higher in the random sample than in the other two.

In the “Culture” representation, no differences were observed with the chi-square test; however, in the online survey, this representation was closer to the third word mentioned, unlike in the survey carried out with ballots. On the other hand, in the random sample, this representation was positioned closer to the fourth word mentioned. In the “Craft” representation, the APs were similar in the sample obtained with the online survey and the randomly selected one; however, in the study with ballot papers, this representation was positioned close to the first word mentioned. Likewise, in the three study samples, the CSIs were similar.

“Family and State of Mexico” were representations with similar CSIs; however, regarding the APs, there were differences. For example, the AP of “Family” in the random sample was related to the second word mentioned among the participants. “Gastronomy and Tourism” presented similar APs and CSIs (with slight variations). These results corroborate that the representations tend to have specific stability and cognitive importance in the two types of surveys. Furthermore, the random sample corroborated the projective methods’ stability, since the APs and CSIs were similar to the other two samples. 

### 3.2. Structural Approach of Social Representations towards Traditional Mexican Cheeses

**Figure 1:**
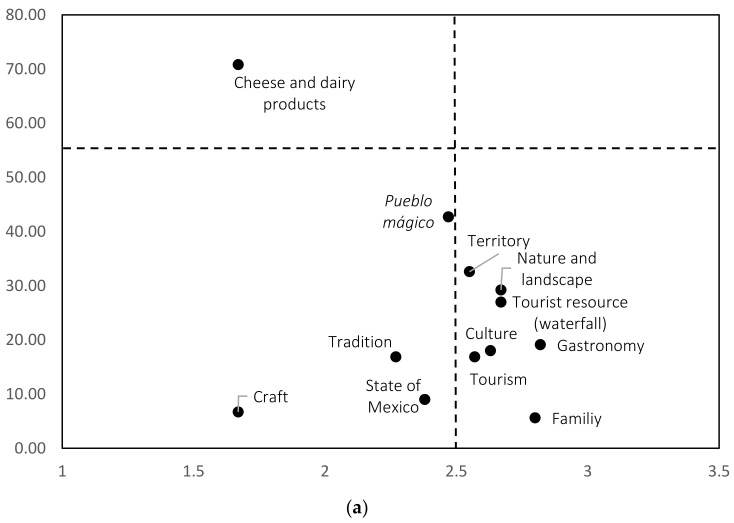

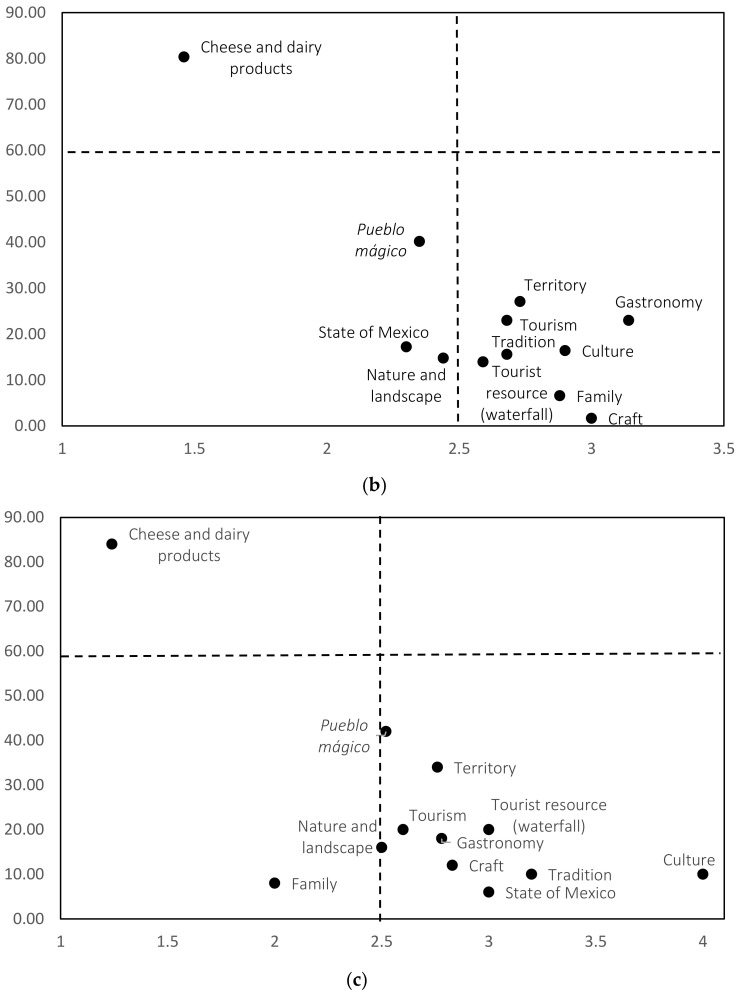
Prototype maps of the three study samples. (**a**) Paper ballots. (**b**) Online survey. (**c**) Random sample.

The second objective of interest in this research was to study, using the structural approach of social representations, the stability and validity of the object of representation, by identifying the central core and the peripheral areas. Therefore, prototype maps were constructed, identifying the four zones in the three study samples (Figure 1).

In the three maps, the representation “Cheese and dairy products” was positioned in the central core, corroborating the above results. In other words, there is a direct connection between the territory and traditional foods; in this case, the dairy products made in the region. The three study samples reported stability and validity towards the object of representation. However, the APs of this representation were close to the first word mentioned in the random sample and the online survey.

In the three maps, no representations were observed in the first periphery (upper-right quadrant). The representation “Pueblo mágico” is worth noting, which was positioned in the zone of contrasting elements in the online and with ballots surveys. However, in the random sample, this representation was very close to this area. The representations “Culture, Gastronomy, Tourism, Tourist resource (waterfall) and Territory” were positioned in the second periphery of the three maps. In general, it can be observed that most of the representations have similar configurations in the four zones of the prototype map. However, the differences identified were in the peripheral areas, which suggests that these areas are more sensitive to change and strongly depend on the sociocultural aspects of the study samples; likewise, their function is to protect the central core, as demonstrated in this research. 

### 3.3. Cluster Analysis and MFA

**Figure 2:**
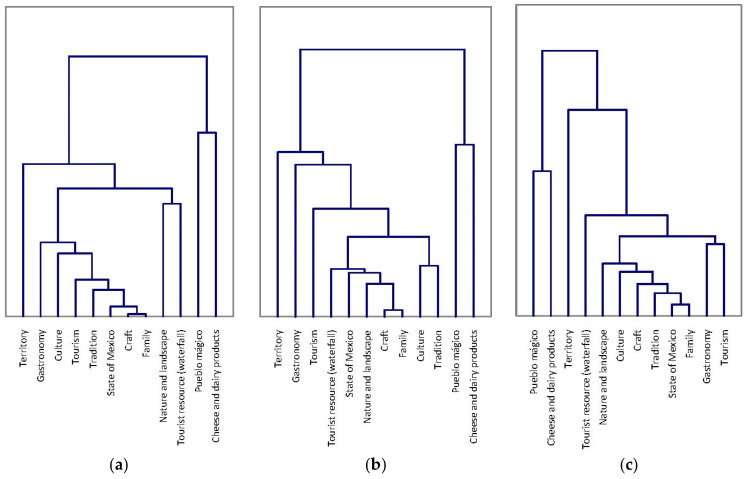
Cluster analysis based on distance matrices from the three study samples. (**a**) Survey with ballot papers. (**b**) Online survey. (**c**) Random sample.

On the other hand, cluster analysis was performed with the distance matrices obtained from the three study samples. Two clusters were identified in each study, which can be observed in Figure 2. The dendrograms have similarities regarding the configuration of the clusters. The first cluster grouped the representations “Cheese and dairy products” and “Pueblo mágico” together, which are crucial for the representation object, and this grouping is consistent with the results identified in the prototype maps, since the first representation was positioned in the central core, while the second had a high mention frequency that allowed it to position itself in the contrasting elements zone (except for the random sample), very close to the central core. The other representations were agglomerated in the second cluster and reflect the heterogeneity of the representations, which protects the central core.

**Figure 3:**
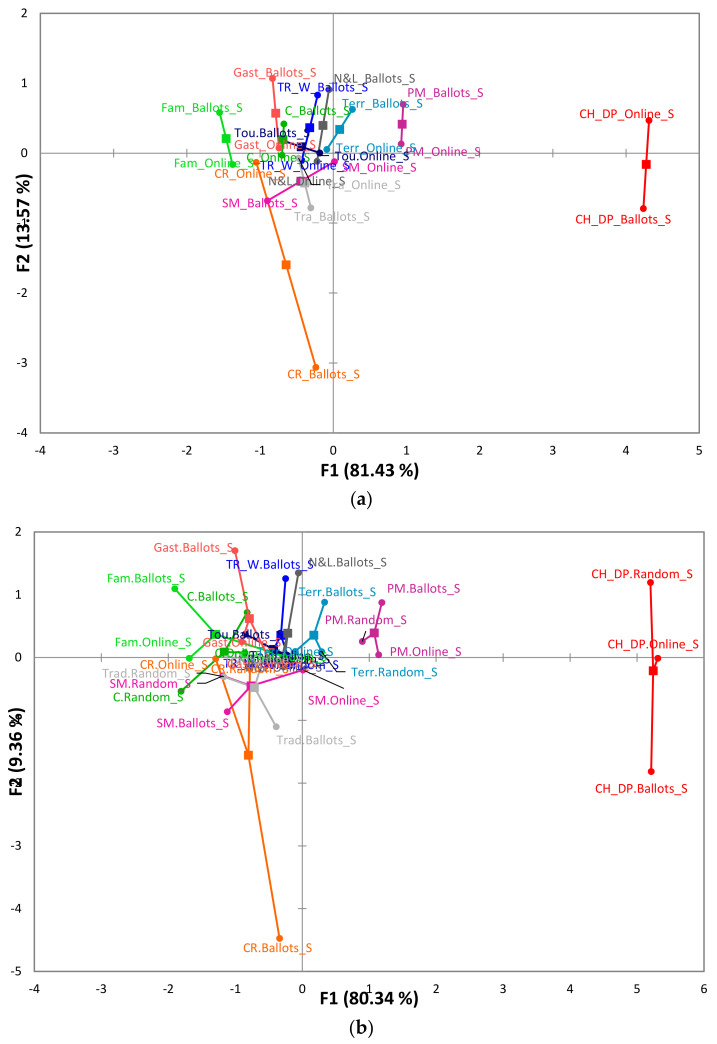
MFA of the social representations identified in the three study samples. (**a**) MFA of the social representations of online and ballot paper surveys. (**b**) MFA of the social representations of the three study samples. **Note:** all representations identified in the two studies were abbreviated as follows: cheese and dairy products (CH_DP); tourist resource (TR_W); culture (C); pueblo mágico (PM); nature and landscape (N&L); craft (CR); tourism (Tou); family (Fam); State of Mexico (SM); gastronomy (Gast); territory (Terr); tradition (Tra).

The map obtained from the MFA shows the similarities of the results obtained from the two types of surveys (ballots and online) with the WA projective technique. The first factor explained 81.43% of the variance, while the second explained 13.57%. The “Craft” representation was the only representation with more significant differences, possibly due to the average position that this representation occupied in each study, which was closer to the first word mentioned in the survey with ballots. The RV coefficient corroborates the results obtained from the MFA map, since a value of 0.87 was obtained, indicating that the representations from the projective methods are similar and present stability and validity for both surveys (Figure 3a).

Finally, we decided to carry out a second MFA (Figure 3b) to establish the similarity of the representations of the three samples studied. This second analysis explained 89.7% of the variance of the three blocks of variables used. The first factor explained the most significant amount of information (80.34%). Furthermore, the “Craft” representation again presented the greatest variability, possibly due to the difference in the AP of the ballot paper survey concerning the online and the random sample. However, the RV correlation coefficient was ≥0.80 among the three study samples, which suggests that this coefficient is a measure that compares the results obtained and allows to indicate the reliability and validity of the information presented in this research using projective methods.

## 4. Discussion

The primary purpose of this research was to study the stability and validity of the information obtained from projective methods, particularly with the WA technique, with the following two types of surveys: face-to-face surveys with ballots and online surveys [16]. For both studies, the same stimuli were used to extract information from the participants, using a case study of a territory characterized by the production of traditional cheeses in Central Mexico [63,64]. In addition, a random sample (n = 50) of both studies was selected to corroborate the stability and validity of the identified social representations.

To our knowledge, this is one of the first studies to establish, despite the qualitative nature of the information obtained with projective methods in the study of food consumer behavior, the stability and validity of the obtained data. Twelve representations were obtained in the three study samples. In most of the representations (n = 11), no statistically significant differences were found according to the chi-square test (*p* > 0.05), despite differences in the sociodemographic characteristics (age and educational level) and frequency of consumption of the samples studied.

Viana et al. [16] highlight the importance of comparing and analyzing the validity of the information obtained from projective methods with online and ballot paper surveys. Gambaro [12] establishes that one of the challenges of projective techniques is the validity and reliability of the information obtained with these methods; that is, consistent and reliable information is obtained from repetitive studies regardless of the characteristics of the population. Recently, Rojas-Rivas et al. [11] reviewed four studies (including cross-cultural studies) that use the WA technique to identify consumers’ perception of traditional foods, establishing that although the studies were carried out in different spatial, temporal, cultural, and sociodemographic conditions, ten categories were identified that indicate stability in the construction or representation of consumers’ perceptions towards traditional foods.

However, it is noteworthy that culture is a factor that influences the stability and validity of information from projective methods. In cross-cultural studies that use the WA technique, it has been shown that each population or culture has a particular way of representing food or food concepts [7,51,65]. Despite cultural differences, some representations show stability; for example, Son et al. [51] did not identify statistical differences in some of the representations of consumers from Korea, Japan, Thailand, and France towards rice, establishing that it is a primary food product, associated with economic, family and traditional aspects in the four cultures. Rojas-Rivas et al. [11] indicate that cross-cultural studies provide valuable information on the validity and stability of the information obtained from the WA technique.

Likewise, the representations “Cheese and dairy products, Territory, Culture, Gastronomy and Family” are consistent with the results reported by Soares et al. [43], who studied the perception of consumers towards Coalho cheese in Brazil and identified that it is a product associated with culture, particularly in the northeastern region, where consumers relate it to ingredients, forms of production, an accompaniment of typical dishes (gastronomy) and family. Although sensory aspects are the main factors that influence consumer perceptions of different types of cheese [43,44,46,66], other factors influence perception, such as packaging, production regions, places, consumption occasions, security aspects, price, processing technology or attitudes and feelings [46,66]. In the case of this research, although some cheese produced in the region was not used as a stimulus to extract consumer associations, it was observed that not only is the territory associated with cheeses and dairy products from the area, but also with cultural aspects, nature and landscape and tourist activities, among other extrinsic aspects that are associated with the territory in the representations of consumers.

On the other hand, the structural approach has proven to be a theoretical tool that contributes to the study of the validity and stability of the information obtained with projective methods, since the prototype maps showed a similar configuration in the organization of the representations in the three study samples. Several studies have used this theoretical approach to study how different population groups construct concepts or perceive characteristics associated with food [22,23,24,25,26,67]. However, it had not been used to compare results obtained from ballot and online surveys. 

The representation “Cheese and dairy products” was positioned in the central core of the three maps. The above finding suggests that consumers associate traditional foods with the territory of production, since this representation is firmly anchored in the collective memory of the study groups. Rodrigues et al. [22] establish that “terroir” is the only stable element in the representation of the minerality of wine between consumers and winemakers. According to other authors [20,22,53], the central core maintains the ideas, knowledge, or information that give meaning to the object of representation over time. The central core is based on a social group’s memory and historicity. 

It is noteworthy that the representation “Pueblo mágico”, which emerged near the central core in the three study samples, even in the HCA, formed a cluster together with “Cheese and dairy products”. In this context, since 2015, the municipality of Aculco has been integrated into the Mexican tourism program called “Pueblos mágicos”, which aims to promote tourism at a national and international level, since the municipalities or localities that belong to this program are characterized by having symbolic, historical and natural attributes [68]. These results suggest that the representation of the territory is linked to traditional foods and tourist aspects, so there could be great opportunities to take advantage of the territory through gastronomic tourism activities, since cheeses have acquired a more significant role as tourist attractions in different destinations [69,70].

The other representations were positioned in the peripheral areas of the prototype maps. These areas represent the heterogeneity of the object of representation, since they are more sensitive to change and the incorporation of new terms in the collective memory of a social group; however, their function is the protection of the central core [20]. It is essential to mention that in most of the representations, the APs and CSIs were similar, reinforcing our results regarding the validity and stability of the information in the case study.

Finally, some considerations derived from this research could be used for future studies at the methodological level. First, it is essential to highlight the contrasting of the representations (words grouped into categories) by the three experts, which guarantees a consensus on the name and frequency of each category/representation [13]. In addition, the minimum frequency criterion considered the representations as part of the analysis [11]. Second, the triangulation process was essential and included using different methods, statistical tools, and theoretical perspectives to better understand the object of study [71]. In this case, the structural approach of social representations and cognitive tools such as the CSI were combined. Third, the stability and validity of the information obtained with projective techniques, in conjunction with the statistical methods used, was confirmed, since in eleven representations, no statistical differences were identified with the chi-square test. Likewise, the maps obtained from the MFA and the RV coefficient showed a similar configuration of the representations in the three study samples. 

Generally, the stability of information is evaluated with repetitive studies and under similar conditions in which consistent results are produced [72,73], as reported in this research. However, future studies must investigate the stability and validity of the information obtained with projective methods with other products, stimuli, concepts or food categories. Another limitation of our study is the size of the samples used in both studies, so future research must replicate our findings with larger and more representative samples. It should be noted that the methodology used in this study is the first step to identifying the stability and validity of the information of projective methods and uses a combination of qualitative (structural approach of social representations) and quantitative tools. Therefore, future works could use/incorporate other methods to analyze the stability and validity of projective techniques. Although the RV coefficient was used as a measure of correlation to identify the similarity and consistency in the stability and validity of the results obtained from the three study samples, it is important for future studies to incorporate other validation tools, such as the comparison of the APs and CSIs of social representations. It is recommended to carry out more repetitive studies with food products or concepts, comparing the representations according to personal characteristics (gender, age and income) and consumption patterns. Finally, future works could investigate the validity and stability of the information with other projective methods.

## 5. Conclusions

This preliminary research aimed to study the stability and validity of the information obtained from the projective methods using the WA technique with the case of traditional cheeses from Central Mexico. The frequency of mention in most of the identified representations was not statistically different, which can be corroborated with the APs and CSIs. The prototype maps obtained from the structural approach of social representations made it possible to identify that the representations were similarly configured in the three study samples. Therefore, the results suggest that projective methods provide stable information about a representation object associated with traditional foods or products; in this case, with the cheeses and dairy products of the study region. In addition, the MFA and the RV coefficient allowed us to observe that the results obtained from both surveys indicate stability and validity, given that the RV coefficient showed a strong correlation between the two analyzed matrices; in other words, the results of the social representations were similar with the online and ballot paper surveys. In the case of the random sample, it was possible to observe similarity with the other two surveys according to the RV coefficient.

The results of this research may have various practical implications for future research that seeks to study the validity and stability of information of a qualitative nature, including the use of projective methods. The structural approach of social representations could be helpful in repetitive studies with consumers of different consumption patterns or sociodemographic characteristics in order to understand the organization in cognitive and attitudinal terms of their perceptions of various food products or concepts. In addition, it could be useful to study the stability of the information in longitudinal studies that use projective methods.

## Figures and Tables

**Table 1:** Sociodemographic characteristics of the samples studied from the two surveys: paper ballots and online survey.

	Total	Online n = 122	Paper Ballots n = 89	*p*
**Sex**				
Men	87	50	37	0.931
Woman	124	72	52	
**Age**				
17–25	40	39 (+)	1 (−)	
26–35	71	41	30	**<0.001**
36–45	53	21 (−)	32 (+)	
46 and above	47	21	26	
**Educational level**				
Low	29	0 (−)	29 (+)	
Middle	64	33	31	**<0.001**
High	118	89 (+)	29 (−)	
**Frequency of cheese consumption**				
Frequent	39	30	9	
Regular	137	74	63	**<0.026**
Not frequent	35	18	17	

**Note:** The signs + or − indicate if the observed frequencies were higher or lower than the theoretical frequencies according to the chi-square test (*p* < 0.05).

**Table 2:** Stability and validity of social representations from the three study samples.

	Paper Ballots n = 89	AP	CSI	Online Survey n = 122	AP	CSI	Random Sample n = 50	AP	CSI	*p*
Cheese and dairy products	63 (0.71)	1.67	0.425	98 (0.80)	1.46	0.550	42 (0.84)	1.24	0.678	0.129
Tourist resource (waterfall)	24 (0.27)	2.67	0.101	17 (0.14)	2.59	0.054	10 (0.20)	3.00	0.167	0.061
Culture	16 (0.18)	2.63	0.068	20 (0.16)	2.90	0.057	5 (0.10)	4.00	0.025	0.444
Pueblo mágico	38 (0.43)	2.47	0.173	49 (0.40)	2.35	0.171	21 (0.42)	2.52	0.166	0.929
Nature and landscape	26 (0.29)	2.67	0.114	18 (0.15)	2.44	0.060	8 (0.16)	2.50	0.064	0.025
Craft	6 (0.07)	1.67	0.040	8 (0.07)	2.88	0.023	6 (0.12)	2.83	0.042	0.438
Tourism	15 (0.17)	2.57	0.061	28 (0.23)	2.68	0.086	10 (0.20)	2.60	0.077	0.552
Family	5 (0.06)	2.80	0.020	2 (0.02)	3.00	0.005	4 (0.08)	2.00	0.040	0.121
State of Mexico	8 (0.09)	2.38	0.038	20 (0.16)	2.30	0.071	3 (0.06)	3.00	0.020	0.093
Gastronomy	17 (0.19)	2.82	0.068	28 (0.23)	3.14	0.073	9 (0.18)	2.78	0.065	0.691
Territory	29 (0.33)	2.55	0.128	33 (0.27)	2.73	0.099	17 (0.34)	2.76	0.123	0.561
Tradition	15 (0.17)	2.27	0.074	19 (0.16)	2.68	0.058	5 (0.10)	3.20	0.031	0.533

**Note:** The relative frequencies of each representation are in brackets.

## Data Availability

The data presented in this study are available upon request from the corresponding author.

## Appendix A

**Table A1:** Main words of the social representations identified in the two studies.

	Survey with Paper Ballots (n = 89)	Online Survey (n = 122)
	*Main words of each representation*	*Main words of each representation*
Cheese and dairy products	Cheese, cheeses, milk, Oaxaca cheese	Cheese, cheeses, milk, cows, Oaxaca cheese
Tourist resource (waterfall)	Waterfall (s), water, its water	Waterfall (s), water, beach
Culture	Culture, history	Culture, history, Mexico, customs, root, historic
Pueblo mágico	Town, pueblo mágico, magic, typical town	Pueblo mágico, town (s), magic, typical town
Nature and landscape	Nature, landscape (s), beautiful, beautiful place	Nature, natural place, place, landscape, beautiful
Craft	Craft, crafts	Craft, crafts
Tourism	Tourism, tourist spot, travel, visit	Tourism, tourist spot, holidays, travel, visit, trip, hotel
Family	Family, families	People, family
State of Mexico	Municipality, State of Mexico	State of Mexico, municipality, north of the state of Mexico
Gastronomy	Gastronomy, food, foods, taste, quesadillas	Gastronomy, food, delicious food, taste, tasty
Territory	Charros, charrería, place, rural, livestock, cattle	Region, Otomies, cattle, livestock, rural, farm
Tradition	Tradition, traditions	Tradition (s), traditional
